# Differential Effects of TipE and a TipE-Homologous Protein on Modulation of Gating Properties of Sodium Channels from *Drosophila melanogaster*

**DOI:** 10.1371/journal.pone.0067551

**Published:** 2013-07-18

**Authors:** Lingxin Wang, Yoshiko Nomura, Yuzhe Du, Ke Dong

**Affiliations:** Department of Entomology, Genetics and Neuroscience Programs, Michigan State University, East Lansing, Michigan, United States of America; Virginia Commonwealth University, United States of America

## Abstract

β subunits of mammalian sodium channels play important roles in modulating the expression and gating of mammalian sodium channels. However, there are no orthologs of β subunits in insects. Instead, an unrelated protein, TipE in *Drosophila melanogaster* and its orthologs in other insects, is thought to be a sodium channel auxiliary subunit. In addition, there are four *TipE*-homologous genes (*TEH1-4*) in *D. melanogaster* and three to four orthologs in other insect species. TipE and TEH1-3 have been shown to enhance the peak current of various insect sodium channels expressed in *Xenopus* oocytes. However, limited information is available on how these proteins modulate the gating of sodium channels, particularly sodium channel variants generated by alternative splicing and RNA editing. In this study, we compared the effects of TEH1 and TipE on the function of three *Drosophila* sodium channel splice variants, DmNa_v_9-1, DmNa_v_22, and DmNa_v_26, in *Xenopus* oocytes. Both TipE and TEH1 enhanced the amplitude of sodium current and accelerated current decay of all three sodium channels tested. Strikingly, TEH1 caused hyperpolarizing shifts in the voltage-dependence of activation, fast inactivation and slow inactivation of all three variants. In contrast, TipE did not alter these gating properties except for a hyperpolarizing shift in the voltage-dependence of fast inactivation of DmNa_v_26. Further analysis of the gating kinetics of DmNa_v_9-1 revealed that TEH1 accelerated the entry of sodium channels into the fast inactivated state and slowed the recovery from both fast- and slow-inactivated states, thereby, enhancing both fast and slow inactivation. These results highlight the differential effects of TipE and TEH1 on the gating of insect sodium channels and suggest that TEH1 may play a broader role than TipE in regulating sodium channel function and neuronal excitability *in vivo*.

## Introduction

Voltage-gated sodium channels are transmembrane proteins that are critical for the initiation and propagation of action potentials in neurons and other excitable cells [1]. Upon membrane depolarization, sodium channels open, resulting in sodium ion influx and further depolarization of the membrane potential. This process is called channel activation, which is responsible for the rapidly rising phase of action potentials. After channel opening, sodium channels inactivate rapidly, within a few milliseconds in a process known as fast inactivation. Fast inactivation plays an important role in the termination of action potentials. Furthermore, in response to prolonged depolarization (seconds to minutes), sodium channels progressively enter into more stable, slow-inactivated states. This process is known as slow inactivation, which is important for regulating membrane excitability, action potential patterns and spike frequency adaptation [2].

Mammalian sodium channels are composed of a pore-forming α subunit and one or more β subunits. Sodium channel α subunits have four homologous domains (I–IV), each containing six transmembrane segments (S1-S6). Mammals have nine α-subunit genes which encode sodium channel isoforms with different gating properties and different expression patterns in various cell types, tissues, and developmental stages, presumably to fulfill unique physiological functions in specific neuronal and non-neuronal cells [1], [3] [4]. Four homologous β subunits (β1-β4) have been identified and characterized [5]. They are small transmembrane proteins that possess an extracellular immunoglobulin (Ig) domain, a single transmembrane segment, and a short intracellular C-terminal domain [6]. β subunits are widely recognized as both channel modulators and cell adhesion molecules [6], [7]. They modulate sodium channel expression and channel gating; and also regulate cell adhesion and migration [6], [7]. A particular β subunit can have variable effects on different sodium channel isoforms. For instance, β2 causes a depolarizing shift in the steady-state inactivation of Na_v_1.2 channels, but has little effect on Na_v_1.3 channels [8], [9]. Different β subunits can also have different effects on a given sodium channel isoform. For instance, β1, β2 and β3 all accelerate fast inactivation kinetics of Na_v_1.8 channels. However, β1 enhances peak sodium current of Na_v_1.8 channels and causes hyperpolarizing shifts in the voltage-dependences of activation and inactivation, whereas β2 and β3 have no effect on peak sodium current and cause depolarizing shifts in the voltage-dependence of activation and inactivation of Na_v_1.8 channels [10].

In contrast to mammals, insects appear to have only a single sodium channel gene that encodes the α-subunit equivalent of mammalian sodium channels [11], [12]. Despite having only a single gene, insects employ alternative splicing and RNA editing to generate many sodium channel variants with different gating and pharmacological properties [12], [13]. Interestingly, there are no orthologs of mammalian β subunit in insects [14]. Instead, a transmembrane protein, TipE, is considered to be an auxiliary subunit of insect sodium channels because it increases the functional expression of insect sodium channels in *Xenopus* oocytes; and *TipE* ^*-*^ mutants exhibit a temperature-sensitive paralytic phenotype, similar to sodium channel mutants [12], [15] [16], [17]. 

Derst and associates identified four *TipE*-homologous genes (*TEH1-4*) in the genome of *Drosophila melanogaster* [18]. TEH1 is expressed in the central nervous system, whereas the transcripts of the other three were also detected in non-neuronal tissues, such as fat body and gut [18]. TEH1-3 proteins have been shown to increase the amplitude of sodium currents of a *Drosophila* sodium channel in *Xenopus* oocytes [18]. TEH1 has also been shown to shift the voltage-dependence of fast inactivation in the hyperpolarizing direction and slow the recovery from fast inactivation of a *Drosophila* sodium channel (different from sodium channel variants in this study) [18]. TipE accelerates the inactivation kinetics of the same *Drosophila* sodium channel [17]. However, the extent of TipE- or TEH1-mediated gating modification and whether their effects are variant-specific remains unclear.

Sodium channels are the primary target of pyrethroid insecticides [19], [20]. Because of the involvement of sodium channel mutations in pyrethroid resistance, intense research has been carried out in the past two decades to functionally express and characterize the effects of pyrethroids on the gating properties of insect sodium channels in *Xenopus* oocytes [12], [21]. Prior to this study, almost all functional and pharmacological analyses of insect sodium channels were conducted by co-expression of insect sodium channels with TipE, and it is not clear whether TipE or TEH1 modulate the action of pyrethroids.

In a previous study, we identified 33 functional *Drosophila* sodium channel (DmNa_v_) splice variants with a wide range of voltage dependences of activation and inactivation [22]. In this study, we used three of these splice variants, DmNa_v_9-1, DmNa_v_22 and DmNa_v_26, to compare the effects of TipE and TEH1 on DmNa_v_ channels. We chose these three variants because in the absence of TipE or TEH1, they generate sufficient currents for electrophysiological analysis, which made it possible to evaluate the gating-modifying effects of TipE or TEH1. In addition, these variants belong to three different splice types and exhibit different functional properties [22], which potentially allows us to determine variant-specific gating modulation by TipE and/or TEH1. Our results show that, like TipE, TEH1 enhanced the expression of sodium currents and accelerated current decay of all three variants. Furthermore, we found that TEH1 extensively modified sodium channel functional properties of all three variants, whereas TipE only modified the gating of one of the variants. TEH1, but not TipE, also reduced DmNa_v_9-1 sensitivity to deltamethrin by reducing the duration of sodium channels in the open state. Our findings raise the possibility that TEH1 may play a broader role in regulating sodium channel gating and neuronal excitability *in vivo*.

## Materials and Methods

### Ethics statement

All animal protocols used in this study were approved by the Institutional Animal Care and Use Committee at Michigan State University.

### *Xenopus* *oocyte* expression system

Oocytes were obtained surgically from female *Xenopus laevis* (Nasco, Ft. Atkinson. WI) and incubated with 1 mg/ml Type IA collagenase (Sigma Co., St. Louis, MO) in Ca^2+^-free ND-96 medium (96 mM NaCl, 2 mM KCl, 1 mM MgCl_2_, and 5 mM HEPES, pH 7.5). Follicle still remaining on the oocytes following digestion was removed with forceps. Isolated oocytes were incubated in ND-96 medium containing 1.8 mM CaCl_2_ supplemented with 50 µg/ml gentamicin, 5 mM pyruvate, and 0.5 mM theophylline [23]. Healthy stage V-VІ oocytes were used for cRNA injection. *TipE/TEH1* cRNA or H_2_O (as control) was injected together with *DmNa*_*v*_ cRNA at a 1:1 ratio.

### Electrophysiological recording and analysis

Methods for two-electrode recording and data analysis were similar to those described previously [24]. The borosilicate glass electrodes were filled with filtered 3 M KCl in 0.5% agarose and had a resistance of 0.5 to 1.0 MΩ. The recording solution was ND-96 recording solution (96 mM NaCl, 2.0 mM KCl, 1.0 mM MgCl_2_, 1.8 mM CaCl_2_, and 10 mM HEPES, pH adjusted to 7.5 with NaOH). Sodium currents were measured with a Warner OC725C oocyte clamp amplifier (Warner Instrument, Hamden, CT) and processed with a Digidata 1440 (Axon Instruments Inc., Foster City, CA). Data were sampled at 50 kHz and filtered at 2 kHz. Leak currents were corrected by p/4 subtraction. pClamp 10.2 software (Axon Instruments Inc., CA) was used for data acquisition and analysis. The maximal peak sodium current was about 2 µA to achieve optimal voltage control by adjusting the incubation time after injection.

The voltage dependence of sodium channel conductance (*G*) was calculated by measuring the peak current at test potentials ranging from −80 mV to +65 mV in 5-mV increments and divided by (*V−V*_rev_), where *V* is the test potential and *V*_rev_ is the reversal potential for sodium ion. Peak conductance values were normalized to the maximal peak conductance (*G*_max_) and fitted with a two-state Boltzmann equation of the form G/G_max_ = [1 + exp(V−V_*1/2*_)/*k*]^−1^, in which *V* is the potential of the voltage pulse, *V*_1/2_ is the voltage for half-maximal activation, and *k* is the slope factor.

The voltage dependence of sodium channel fast inactivation was determined by using 100-ms inactivating pre-pulses ranging from -120 mV to 0 mV in 5 mV increments from a holding potential of −120 mV, followed by test pulses to -10 mV for 20 ms. The peak current amplitude during the test depolarization was normalized to the maximum current amplitude and plotted as a function of the pre-pulse potential. Data were fitted with a two-state Boltzmann equation of the form I/I_max_ = [1 + (exp(V−V_1/2_)/*k*)]^−1^, in which *I* is the peak sodium current, *I*_max_ is the maximal current evoked, *V* is the potential of the voltage pre-pulse, V_1/2_ is the half-maximal voltage for inactivation, and *k* is the slope factor.

The voltage dependence of sodium channel slow inactivation was measured with 60 s conditioning pulses ranging from −100 mV to 0 mV in 10 mV increments, followed by repolarization to a holding potential of -120 mV for 100 ms to remove fast inactivation, and at last a -10 mV test pulse for 20 ms. The peak current amplitude during the test depolarization was normalized to the maximum current amplitude and plotted against the pre-pulse potential. Data were fitted with a two-state Boltzmann equation as above for fast inactivation.

Development of fast inactivation was measured by holding oocytes at -120 mV, followed by a depolarization to -45 mV for 0 to 80 ms, and then a -10 mV test pulse for 20 ms to measure the fraction of sodium current inactivated during the pre-pulse. The peak current during the test pulse was divided by the peak current which has a pre-pulse duration of 0 ms and plotted as a function of duration time of pre-pulse. Time constant (τ) was calculated by fitting the plot with a single exponential decay function.

Recovery from fast inactivation was tested with a conditioning depolarization of -10 mV for 100 ms, which will drive all sodium channels into the fast inactivated state, then repolarization to -70 mV for 0-20 ms followed by a 20-ms test pulse to -10 mV. The peak current during the test pulse was divided by the peak current during the inactivating pulse and plotted as a function of duration time between two pulses. Time constant (τ) of recovery from fast inactivation was calculated by fitting the plot with a single exponential function.

Development of slow inactivation was measured by holding oocytes at -120 mV, followed with a -10-mV pre-pulse depolarization for 0 to 25 s, then repolarization to -120 mV for 100 ms to remove fast inactivation, and a -10 mV test pulse for 20 ms to measure the fraction of sodium current inactivated during the pre-pulse. The peak current during the test pulse was divided by the peak current which has a pre-pulse duration of 0 ms and plotted as a function of duration of pre-pulse. Time constant (τ) was calculated by fitting the plot with an exponential decay function.

Recovery from slow-inactivation was measured by holding oocytes at -120 mV, followed by a pre-pulse to -10 mV for 60 s to drive sodium channels into the slow inactivated state, followed by repolarization to -120 mV for 0 to 30 s, and finally a test pulse to -10 mV for 20 ms. The peak current during the test pulse was divided by the peak current which has a repolarizing duration of 30 s and plotted as a function of duration between the pre and test pulses. Recovery from slow inactivation was well fitted by a double exponential function.

### Measurement of sodium channel sensitivity to deltamethrin

The method for application of deltamethrin in the recording system was identical to that described by Tan et al. [25]. The effect of deltamethrin was measured 10 min after toxin application. Deltamethrin-induced tail currents were recorded with a 100-pulse train of 5 ms step depolarizations from -120 to 0 mV at 66.7 Hz [26]. Additionally, deltamethrin-induced tail currents were measured using a single pulse protocol with a 500-ms step depolarization from -120 mV to -10 mV. The percentage of channels modified by deltamethrin was calculated using the equation *M* = {[*I*_tail_/(*E*_h_ −*E*_Na_)]/[*I*_Na_/(*E*_t_ −*E*_Na_)]}×100 [27], where *I*_tail_ is the maximal tail current amplitude, *E*_h_ is the potential to which the membrane is repolarized, *E*_Na_ is the reversal potential for sodium current determined from the current-voltage curve, *I*_Na_ is the amplitude of the peak current during depolarization before pyrethroids exposure, and *E*_t_ is the potential of step depolarization.

### Chemicals

Deltamethrin was kindly provided by Bhupinder Khambay (Rothamsted Research, Harpenden, UK). Deltamethrin was dissolved in dimethyl sulfoxide (DMSO). The working concentration was prepared in ND-96 recording solution immediately prior to experiments. The concentration of DMSO in the final solution was <0.5%, which had no effect on the function of sodium channels.

### Statistical analysis

Results are reported as mean ± SEM. Statistical significance was determined by using one-way analysis of variance (ANOVA) with Scheffe’s post hoc analysis, and significant values were set at *p*<0.05.

## Results

### TipE and TEH1 increase the peak sodium current and accelerate the current decay of all three DmNa_v_ variants

**Figure 1:**
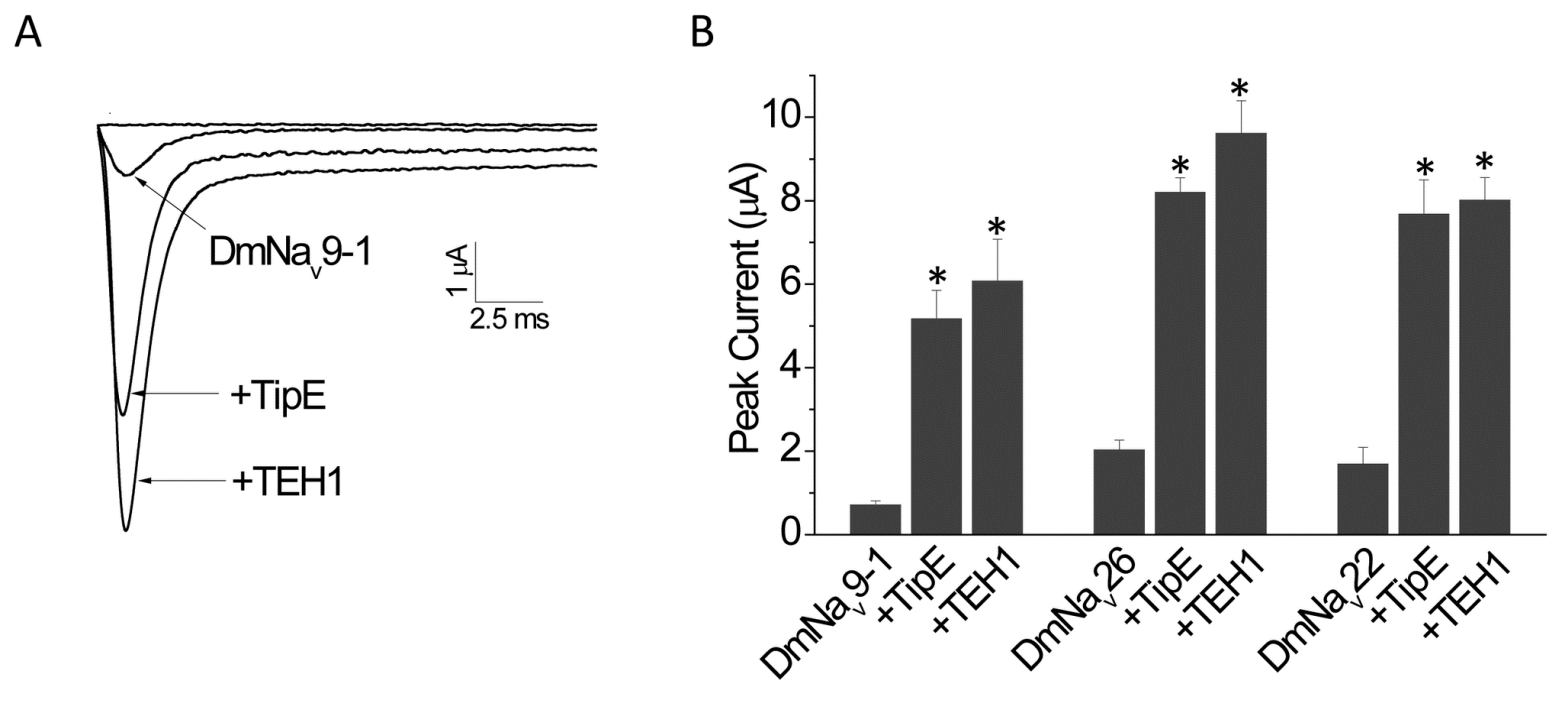
Modulatory effects of TipE and TEH1 on peak sodium currents of DmNa_v_9-1, DmNa_v_22, or DmNa_v_26 channels. (A) Representative traces of peak sodium currents from oocytes expressing DmNa_v_9-1, DmNa_v_9-1+TipE, and DmNa_v_9-1+TEH1 sodium channels. Note that the sodium current of the DmNa_v_9-1 channel possesses a non-inactivating component, known as persistent current (10% of the maximal transient peak current). Both TipE and TEH1 enhanced the persistent current. However, the persistent current remained to be about 10% of the maximal transient peak current. (B) Both TipE and TEH1 significantly increased peak sodium currents of all three Para sodium variants tested: DmNa_v_9-1, DmNa_v_26, and DmNa_v_22, but there was no significant difference between the effects of TipE and TEH1. Sodium currents were recorded 48 hours after cRNA injection. Sodium currents were recorded by a step depolarization to from -80 to 65 mV in 5mV increments with a holding potential of -120 mV. Data are presented as means ± SEM for 12-15 oocytes. * indicates a significant difference compared to peak of DmNa_v_ channel only using one-way ANOVA with Scheffe’s post hoc analysis (*p*<0.05).

To compare the expression of sodium current in *Xenopus* oocytes, equal amounts of cRNA synthesized *in vitro* from the DmNa_v_9-1, DmNa_v_22, or DmNa_v_26 plasmids were injected into oocytes with or without *TipE* or *TEH1* cRNA. Sodium currents were recorded 48 hours after injection by step depolarizations to a series of voltages ranging from -80 mV to +25 mV in 5-mV increments. Both TipE and TEH1 increased the amplitude of peak sodium current of all three variants by 4 to 9 fold (Figure 1A and B).

**Figure 2:**
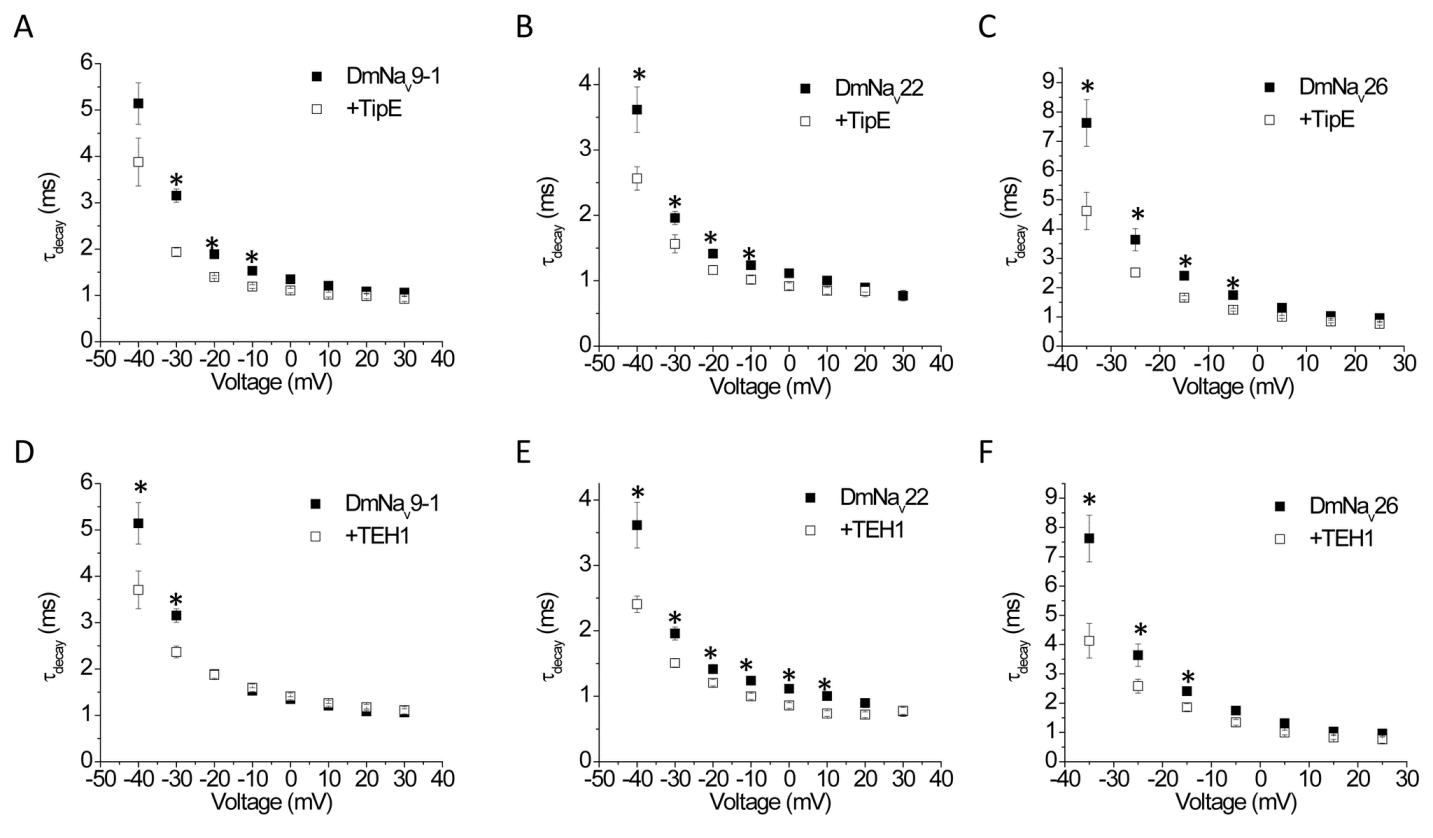
Enhancement of sodium current decay by co-expression of TipE or TEH1 with DmNa_v_ channel variants. Sodium current decay in DmNa_v_9-1, DmNa_v_22, or DmNa_v_26 co-expressed with TipE (A, B, C, respectively) or TEH1 (D, E, and F, respectively). The decay of sodium current was fitted by a single exponential to generate time constants of current decay (τ_decay_). Each data point represents mean ± SEM for 12-20 oocytes. * indicates a significant difference compared to that of DmNa_v_ channel only (*p*<0.05).

We then measured the effect of TipE and TEH1 on the decay of sodium currents for all three variants. Current decay was well fitted by a single exponential with or without TipE or TEH1. TipE slightly but significantly increased the rate of current decay of all three variants between -40 mV and -5 mV (Figure 2A–C). Similarly, TEH1 also slightly accelerated current decay for all three variants, but over different voltage ranges (Figure 2D–F). TEH1 affected the current decay of DmNa_v_22 channels over a broader voltage range (between -40 mV to 10 mV) than the other two variants which were affected between -40 mV and -30 mV for DmNa_v_9-1, and -40 mV and -20 mV for DmNa_v_26 (Figure 2D–F).

### Differential effects of TEH1 and TipE on the voltage-dependence of activation and fast inactivation

**Figure 3:**
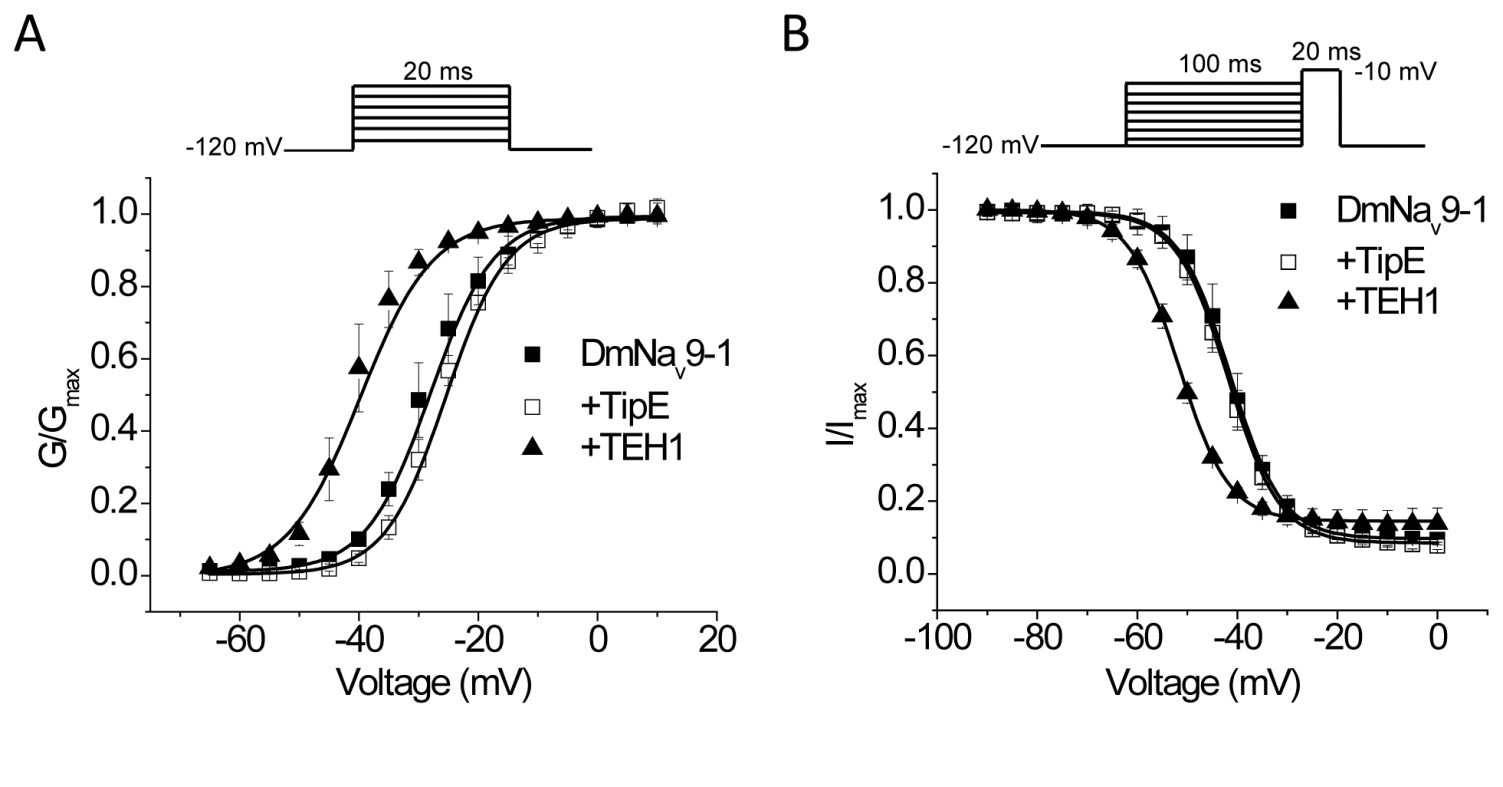
Effects of co-expression of TipE or TEH1 on the voltage-dependence of activation and fast inactivation of DmNa _v_9-1 channels. (A) Voltage-dependences of activation. (B) Voltage-dependence of fast inactivation. Data were fitted with a two-state Boltzmann equation and fitting parameters are shown in Table 1. Data points are shown as mean ± SEM. Recording protocols are indicated and the details of the protocols and data analysis are described in the Materials and Methods.

Co-expression of DmNa_v_9-1 with TEH1 induced a 12-mV hyperpolarizing shift in the voltage dependence of activation (Figure 3A and Table 1). Similarly, significant hyperpolarizing shifts were also detected with co-expression of TEH1 with DmNa_v_26 or DmNa_v_22 (Table 1). However, co-expression of TipE did not modify the voltage dependence of activation of any variants (Figure 3A and Table 1).

**Table 1:** Gating properties of DmNa_v_ variants with or without TipE or TEH1.

		Activation			Fast Inactivation			Slow inactivation	
		V_1/2_ (mV)	*k* (mV)		V_1/2_ (mV)	*k* (mV)		V_1/2_ (mV)	*k* (mV)
DmNa_v_9-1		-29.0 ± 1.1	5.5 ± 0.7		-41.4 ± 0.8	4.8 ± 0.2		-44.5 ± 1.8	5.6 ± 0.8
DmNa_v_9-1 + TipE		-26.2 ± 0.5	5.2 ± 0.2		-42.1 ± 0.7	5.1 ± 0.1		-44.9 ± 1.4	5.5 ± 0.2
DmNa_v_9-1 + TEH1		-41.1 ± 1.1*	4.6 ± 0.2		-51.7 ± 0.6*	5.0 ± 0.1		-60.2 ± 0.5*	5.0 ± 0.3
DmNa_v_26		-25.7 ± 0.2	3.4 ± 0.3		-34.1 ± 0.1	4.1 ± 0.2		-45.6 ± 0.2	5.0 ± 0.3
DmNa_v_26 + TipE		-24.7 ± 0.6	3.4 ± 0.4		-40.8 ± 0.4*	4.2 ± 0.2		-44.1 ± 0.7	4.5 ± 0.4
DmNa_v_26 + TEH1		-33.6 ± 0.6*	3.1 ± 0.4		-42.6 ± 0.6*	4.6 ± 0.1		-50.6 ± 0.3*	4.5 ± 0.5
DmNa_v_22		-26.7 ± 1.1	5.8 ± 0.5		-40.7 ± 0.6	4.8 ± 0.1		-49.2 ± 0.2	3.7 ± 0.2
DmNa_v_22 + TipE		-25.1 ± 1.2	7.2 ± 0.5		-38.9 ± 0.5	4.8 ± 0.2		-45.5 ± 0.8	5.3 ± 0.2
DmNa_v_22 + TEH1		-32.1 ± 0.7*	6.2 ± 0.3		-48.3 ± 0.6*	5.2 ± 0.1		-57.9 ± 0.7*	4.8 ± 0.1

Data represent mean ± SEM for 12-20 oocytes. DmNa_v_ 9 1, DmNa _v_ 26, and DmNa_v_ 22 are treated as control of each group.

* Significantly different from that of DmNa_v_ channel only using one-way ANOVA with Scheffe’s post hoc analysis (*p*<0.05).

Co-expression of DmNa_v_9-1 with TEH1 induced a 9-mV hyperpolarizing shift in the voltage-dependence of fast inactivation compared with DmNa _v_9-1 alone (Figure 3B and Table 1). Similarly, co-expressing TEH1 with DmNa_v_26 or DmNa_v_22 also significantly shifted the voltage dependence of fast inactivation in the hyperpolarizing direction (Table 1). TipE did not alter the voltage dependence of fast inactivation of DmNa_v_9-1 or DmNa_v_22 channels, but caused a significant 6-mV hyperpolarizing shift in the voltage dependence of fast inactivation of DmNa_v_26 channels (Table 1), indicating that TipE has a variant specific effect on the voltage dependence of fast inactivation.

### Effect of TipE and TEH1 on the sensitivity of DmNa_v_9-1 channels to deltamethrin

**Figure 4:**
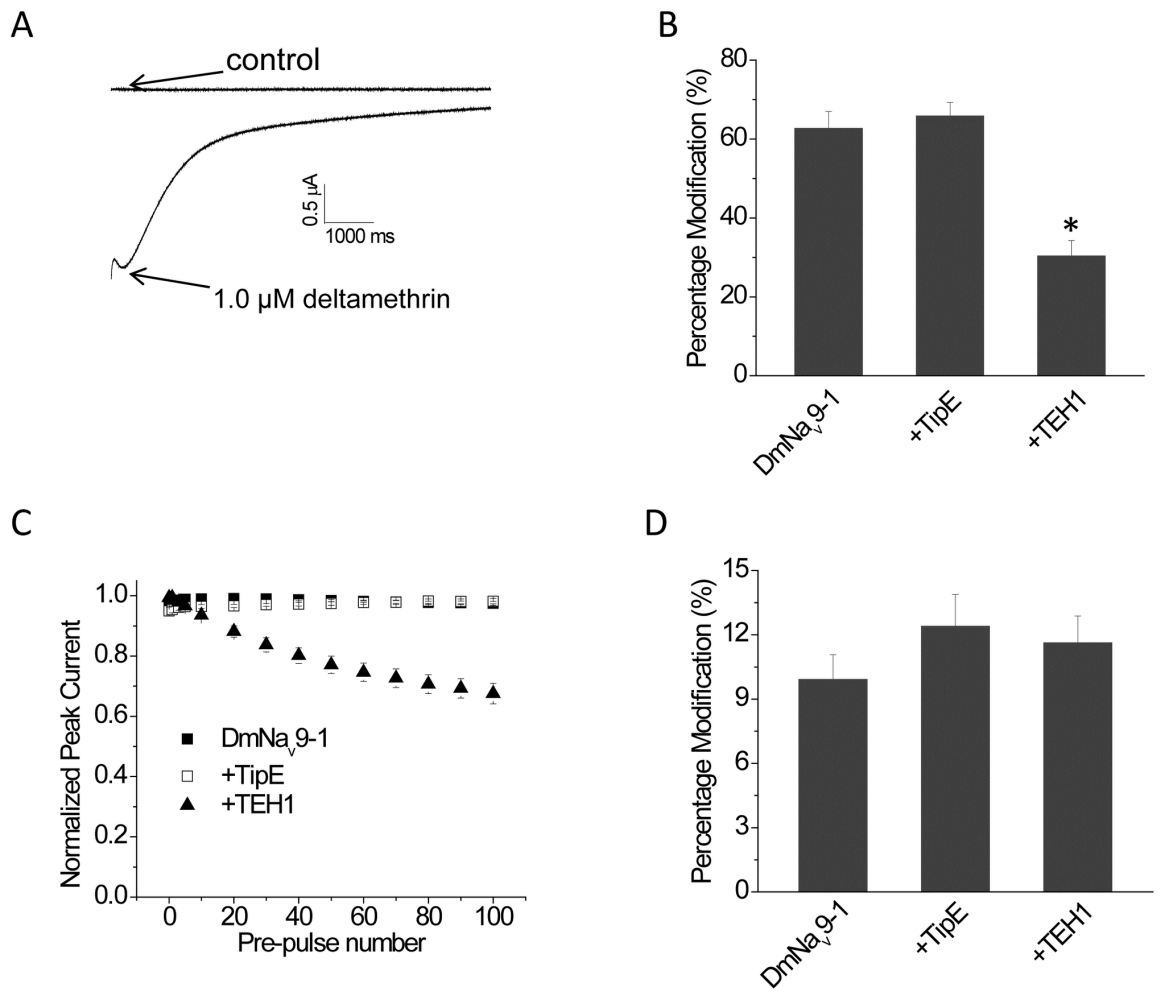
Sensitivity of DmNa _v_9-1 to deltamethrin is modulated by co-expression of TEH1. (A) A representative tail current induced by 1 µM deltamethrin. (B) Percentage of channel modification by deltamethrin (multiple-pulse test). Tail currents were elicited by a 66.7-Hz train of 100 5-ms depolarization from -120 to 0 mV. (C) Co-expression of TEH1 significantly reduced the stability of DmNa_v_9-1 peak sodium current under repeated conditioning depolarizations. Sodium currents were recorded during 20-ms step depolarizations from -120 mV to -10 mV after 0-100 conditioning pulses (5-ms pulses from -120 mV to 0 mV at 66.7 Hz). (D) Percentage of channel modification by deltamethrin (single-pulse test). Tail currents were elicited by a 500 ms depolarization from -120 mV to 0 mV. All data are shown as mean ± SEM for 9-15 oocytes. * indicates significant difference compared to the DmNa_v_9-1 channel using one-way ANOVA with Scheffe’s post hoc analysis (*p*<0.05).

Deltamethrin, a pyrethroid insecticide, induces a slowly decaying tail current associated with repolarization in voltage clamp experiments [26]. To determine whether TipE and TEH1 differentially modulate the activity of deltamethrin, we used a train of depolarizing pulses to elicit deltamethrin-induced tail current in oocytes expressing DmNa_v_9-1 with or without TipE or TEH1. At 1 µM, deltamethrin induced a large tail current (Figure 4A), which can be quantified as the percentage of channel modification by deltamethrin using the method developed by Tatebayashi and Narahashi [27]. Co-expression of TEH1 with DmNa_v_9-1 channels significantly reduced the percentage of channels modified by deltamethrin (Figure 4B), whereas TipE had no effect on channel sensitivity to deltamethrin (Figure 4B).

Derst et al. [18] reported that the recovery from inactivation of a *Drosophila* sodium channel variant was slowed by TEH1. We hypothesized that, in the presence of TEH1, the trains of depolarizing pulses used in our study to evaluate the effect of deltamethrin may reduce the availability of open channels, thereby, reducing the gating modification by deltamethrin. To test whether changes in gating caused by TEH1 were responsible for the reduced channel sensitivity to deltamethrin, we examined the effect of the multiple depolarizing-prepulses on the stability of peak sodium current in channels co-expressed with TipE or TEH1. The peak current remained unchanged in oocytes expressing DmNa_v_9-1 channels alone or with TipE, whereas the peak sodium current was gradually reduced after each conditioning pulse in oocytes coexpressing DmNa_v_9-1channels with TEH1 (Figure 4C). These results support the hypothesis that the reduced deltamethrin sensitivity of DmNa_v_9-1 channels in the presence of TEH1 is likely caused by reduced availability of open channels. We then examined the effect of deltamethrin without the conditioning pulsesand found that the percentage of channel modification by deltamethrin was not altered by either TEH1 or TipE (Figure 4D).

### Co-expression of TEH1 with DmNa_v_9-1 significantly enhanced entry into and stability of the fast-inactivated state

**Figure 5:**
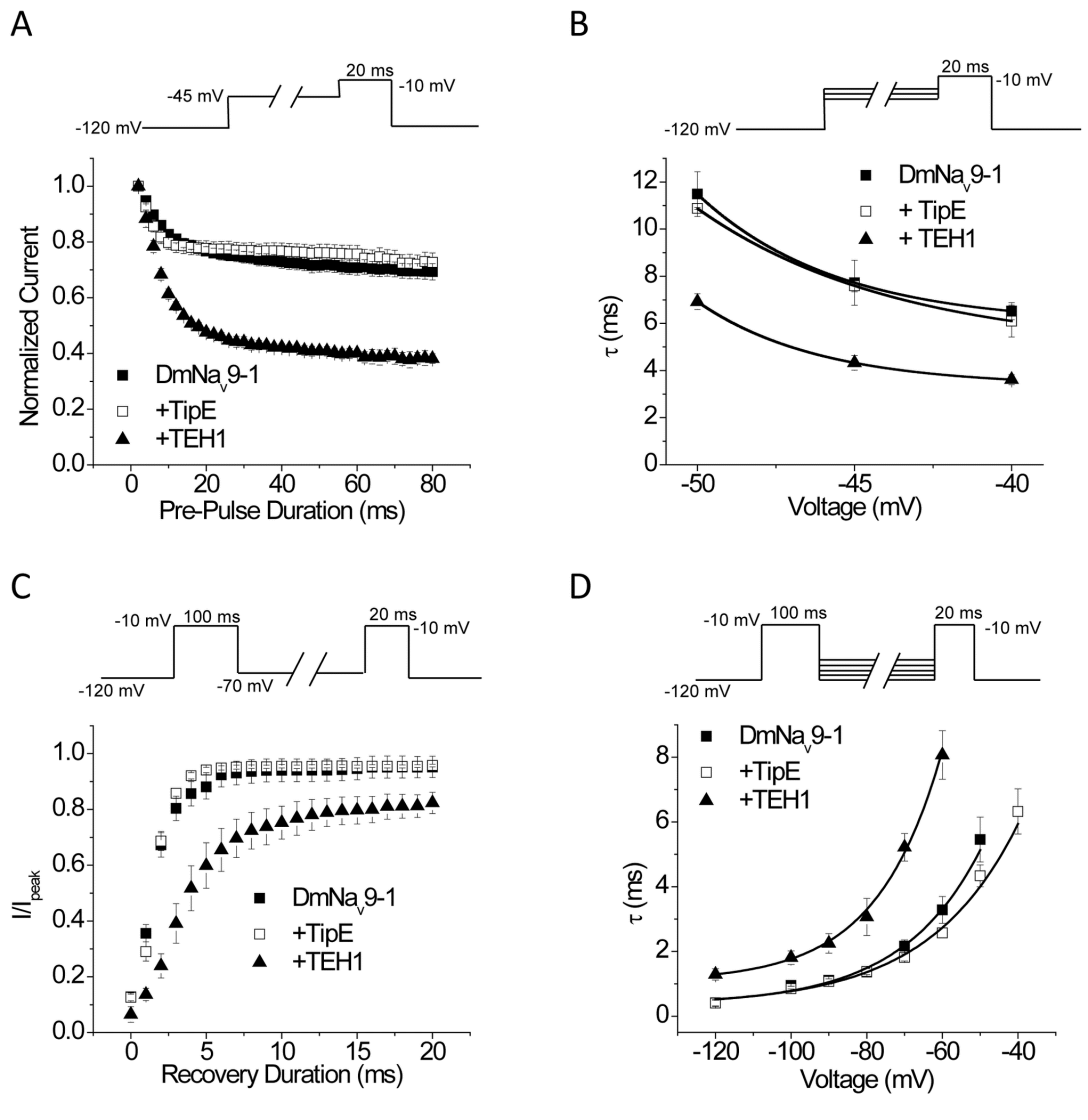
Effects of co-expression of TipE or TEH1 on entry into or recovery from fast inactivation of DmNa_v_9-1 channels. (A) Time course of development of fast inactivation with a pre-pulse of -45 mV. (B) τ values of development of fast inactivation under different pre-pulse voltages. τ values were determined by fitting time course of the development of fast inactivation with a single exponential decay (n ≥ 12). (C) Recovery from fast inactivation with a repolarizing voltage of -70 mV. (D) τ values of recovery from fast inactivation at different repolarizing voltages. τ values were calculated by fitting recovery from fast inactivation data from different repolarizing voltages by a single exponential function (n≥ 10). Recording protocols are indicated and the details of the protocols and data analysis are described in the Materials and Methods.

The results above prompted us to further characterize the effects of TEH1 and TipE on inactivation gating kinetics, particularly the development of and recovery from fast-inactivation. Figure 5A shows the time course of the development of fast inactivation at the pre-pulse voltage of -45 mV for DmNa_v_9-1 alone or co-expressed with TipE or TEH1. Co-expression of TEH1 with DmNa_v_9-1 greatly enhanced entry of DmNa_v_9-1 channels into the fast inactivated state compared with that of DmNa_v_9-1 alone or the combination of DmNa_v_9-1 and TipE (Figure 5A). In addition, the accelerated entry into fast inactivation by TEH1 was observed at all three pre-pulse voltages tested (Figure 5B).

Co-expressing DmNa_v_9-1 with TEH1 greatly inhibited the recovery of DmNa_v_9-1 channels from fast inactivation all repolarization voltages tested (Figure 5C and D). In contrast, co-expression of DmNa_v_9-1 with TipE had no effect on the recovery from fast inactivation (Figure 5C, and D).

### Co-expression of TEH1 with DmNa_v_9-1 inhibited recovery from slow inactivation

**Figure 6:**
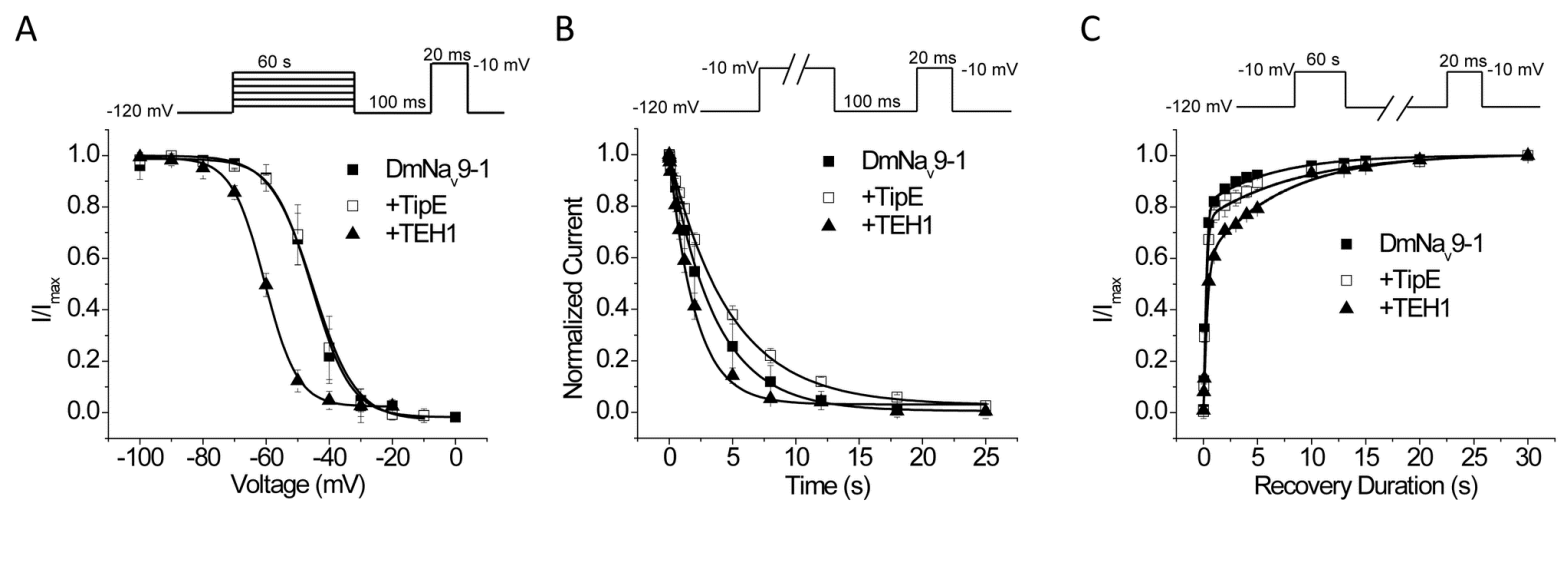
Co-expression of TEH1 inhibits recovery from slow inactivation of DmNa_v_9-1 channels. (A) Voltage-dependence of slow inactivation. (B) Time course of development of slow-inactivation. Development of slow inactivation was fitted by an exponential decay and the parameters are summarized in Table S1. (C) Time course of recovery from slow-inactivation. Recovery from slow inactivation was fitted by a double exponential function and the parameters are summarized in Table 2. Recording protocols are indicated and the details of the protocols and data analysis are described in the Materials and Methods.

In addition to fast inactivation, sodium channels undergo slow inactivation which plays important roles in regulating firing frequency and pattern in response to sustained stimuli [2]. Therefore, we examined the effects of TEH1 and TipE on the voltage-dependence of slow inactivation, the rate of entry into the slow-inactivated state, and recovery from slow inactivation of DmNa_v_9-1channels. Co-expression of DmNa_v_9-1 with TEH1 induced a significant 16-mV hyperpolarizing shift compared with DmNa_v_9-1 alone (Figure 6A and Table 1). TEH1 also induced significant hyperpolarizing shifts in voltage-dependence of slow inactivation in DmNa_v_26 and DmNa_v_22 channels (Table 1). In contrast, TipE had no effect on the voltage-dependence of slow inactivation in any of three variants tested (Table 1).

The development of slow inactivation for DmNa_v_9-1 with or without TipE or TEH1 all exhibited a monophasic time course and was well fitted by an exponential decay (Figure 6B and Table S1). We found that neither TipE nor TEH1 significantly alter the development of slow inactivation of DmNa_v_9-1 channels (Figure 6B and Table S1). The rate of recovery from the slow inactivated state for DmNa_v_9-1with or without TipE or TEH1 all followed a biphasic time course (Figure 6C and Table 2). DmNa_v_9-1+TipE recovered from slow inactivation in a manner that was very similar to that of DmNa_v_9-1 alone. However, co-expression with TEH1 significantly slowed the slow component (τ_2_) of recovery and also increased the fraction of the slow component, but did not alter the fast component (τ_1_) of recovery (Figure 6C and Table 2).

**Table 2:** Recovery from slow inactivation of DmNa _v_9-1 with or without TipE or TEH1.

Na^+^ channel	τ_1_	f_1_	τ_2_	f_2_	n
DmNa_v_9-1	0.23 ± 0.02	0.81 ± 0.01	5.10 ± 0.45	0.19 ± 0.01	9
+ TipE	0.24 ± 0.02	0.79 ± 0.01	4.8 ± 0.42	0.21 ± 0.02	8
+ TEH1	0.24 ± 0.03	0.59 ± 0.02*	7.1 ± 0.38*	0.41 ± 0.01*	10

Recovery from slow inactivation was fitted by double exponential function. Data represents mean ± SEM. τ: time constant, f relative fraction, n number of oocytes.

* Significant difference compared with DmNa^v^9-1 channel using one-way ANOVA with Scheffe’s post hoc analysis (*p*<0.05).

## Discussion

While the roles of mammalian sodium channel β subunits in modulating sodium channel activities have been extensively studied, research on auxiliary subunits of insect sodium channels is limited. This is particularly true with respect to how these auxiliary subunits modulate sodium channel gating and toxin pharmacology. In this study, we showed that while both TipE and TEH1 enhanced peak sodium currents and increased current decay, they modulated the gating of DmNa_v_ channels differently. First, TEH1 induced hyperpolarizing shifts in the voltage-dependences of activation, fast inactivation, and slow inactivation of all three DmNa_v_ sodium channel variants examined. In contrast, TipE did not alter these properties of the three variants, with one exception: TipE shifted the voltage-dependence of fast inactivation of DmNa_v_26 channels in the hyperpolarizing direction. Second, TEH1, but not TipE, facilitated entry of sodium channels into fast inactivation and delayed their recovery from both fast and slow inactivation. Our findings therefore suggest distinct roles of TipE and TEH1 in regulating the function of sodium channels and neuronal excitability *in vivo*.

TipE is the first auxiliary subunit of insect sodium channels identified in *D. melanogaster*. *tipE* ^*-*^ mutants exhibit temperature-sensitive paralytic phenotypes [16], [28], suggesting an important role of TipE in regulating neuronal excitability. An earlier electrophysiological study on the activity of embryonic neurons from a *tipE* ^*-*^ mutant has shown that TipE modulates the activity of only certain neurons [29]. The percentage of embryonic neurons from a *tipE* ^*-*^ mutant capable of firing repetitively during a sustained depolarization was significantly reduced [29]. However, only a portion of the *tipE* ^*-*^ neurons was affected [29]. Additionally, a [H^3^] saxitoxin binding study showed that sodium channel density was reduced by about 30% to 40% in head membrane extracts from the *tipE* ^*-*^ mutants compared with wild type flies [30]. Furthermore, whole-cell patch clamp recordings indicated that sodium current density was decreased by about 40% to 60% in dissociated embryonic neurons of *tipE* ^*-*^ mutants [31]. Consistent with these findings, TipE was shown to enhance the peak current of insect sodium channels in heterologous expression ( *Xenopus* oocytes) studies [12], [16] [17],. TipE and TEH1 drastically increased the amplitude of peak current of all three sodium channel variants. These results suggest they increase sodium current density. Such effects may be exerted at the level of channel protein expression and/or channel conductance.

Although TipE accelerated the current decay of all three variants (Figure 2A-C) in our study, the effects on these three variants were not as drastic as that on the variant in Warmke et al. [17],, suggesting that the effect of TipE on inactivation kinetics may be variant-specific. Furthermore, the effects of TipE on sodium channel gating seem to be limited, compared to TEH1, and may also be variant-specific. The voltage dependence of inactivation of only one variant, DmNa_v_26, that we examined was altered in the presence of TipE (Table 1). DmNa_v_26 differs from the other two variants in the exclusion of one optional exon j, and inclusion of one optional exon f and one mutually exclusive exon k, and also contains five scattered amino acid changes which are possibly due to RNA editing. Which unique sequence(s) contributes to this variant-specific effect remains to be determined. It is known that DmNa_v_ and other insect sodium channel transcripts undergo extensively alternative splicing and RNA editing, generating a large collection of sodium channel variants [12], [13]. These variants exhibit unique gating and pharmacological properties [22], [25] [32], [33], [34]. They may be expressed in different tissues and cells to fulfill their unique roles in insect neurophysiology [32]. It is therefore possible that modulation of gating properties of selective DmNa_v_ variants by TipE provides a unique control of neuronal activities in specific neural circuits. On the other hand, extensive modification of sodium channel gating properties by TEH1 raises the possibility that TEH1 plays a broader role than TipE in modulating the gating of potentially diverse sodium channel variants in *Drosophila* . Enhanced fast and slow inactivation by TEH1 could lead to reduced availability of open sodium channels particularly in response to sustained stimulations of various durations, which could decrease firing frequency and alter firing patterns. As we showed, reduced availability of open channels by TEH1 decreased the potency of pyrethroids because pyrethroids preferably act on open sodium channels. It is possible that TEH1, but not TipE, may regulate sodium channel sensitivity to pyrethroids in neurons that encounter repetitive stimulations. It is also intriguing that TEH1 could modulate three different gating properties, activation, fast inactivation, and slow inactivation, which are thought to be controlled by distinct regions of the sodium channel protein [1], [35], [36]. Further characterization of how TEH1 modulates these three gating properties at the molecular level may uncover interconnecting molecular features that are critical for activation, fast inactivation, and slow inactivation of sodium channels.

Our data suggests that TEH1 has similar modulatory effects on DmNa_v_ variants as the β subunits of mammalian sodium channels. Aside from regulating the expression of sodium channels, mammalian β subunits modify the gating properties of sodium channels and modulate the electrical excitability of nerves and muscles [37]. For example, β1 subunits induce depolarizing shifts in the voltage-dependence of activation, fast inactivation, and slow inactivation of Na_v_1.2 channels [38]. Additionally, co-expression of β1 subunits with Na_v_1.7 and Na_v_1.8 sodium channels in *Xenopus* oocytes accelerates current kinetics and produces a hyperpolarizing shift in steady-state inactivation [39], and modulates activation, slow inactivation, and recovery from slow inactivation of Na_v_1.4 channels [40], [41] [42].

Modulation of the function of sodium channel variants by TEH1 indicates a potential functional coupling of sodium channel variants with TEH1. An earlier study has shown that the TEH1 transcript is detected exclusively in the central nervous system (CNS) [18], where DmNa_v_ transcripts are abundantly expressed [43], suggesting potential co-expression of TEH1 and DmNa_v_ in the CNS. However, further biochemical analysis is needed to confirm co-localization and/or direct physical interaction between TEH1 and DmNa_v_ channels *in vivo*. Structurally, TipE or TEH1 are different from mammalian sodium channel β subunits. Both TipE and TEH1 have intracellular N- and C-termini and two membrane segments connected by a large extracellular loop; whereas β subunits are composed of a single transmembrane segment with an extracellular N-terminus and a small intracellular C-terminus. Both extracellular and intracellular domains of mammalian β1 subunits have been shown to be essential for functional modulation of sodium channels [44], [45], [46]. Future molecular analyses are needed to address the molecular mechanism by which TipE and TEH1 modulate the function of insect sodium channels.

In conclusion, we showed that TipE and TEH1 differentially modulate key gating properties of DmNa_v_, even though both TipE and TEH1 enhance the sodium current and accelerate current decay in all DmNa_v_ variants tested. Furthermore, although TipE and TEH1 are structurally different from mammalian sodium channel β subunits, our results show that these proteins appear to be functionally similar. Thus, not only TipE, but also TEH1, may play an important role in regulating neuronal activities in insects. Further understanding the role of TEH1 *in vivo*, including generation and characterization of *TEH1* mutants, is expected to further advance our general knowledge of sodium channel function and neuronal excitability.

## Supporting Information

Table S1 Development of slow inactivation of DmNa_v_9-1 with or without TipE or TEH1. (DOCX)
