# Supplementary material for: Differential Effects of TipE and a TipE-Homologous Protein on Modulation of Gating Properties of Sodium Channels from Drosophila melanogaster
Source: PLoS One. 2013 Jul 18;8(7):e67551. doi: 10.1371/journal.pone.0067551 (PMC3715519; doi:10.1371/journal.pone.0067551)
Supplement: Table S1 — (DOCX) [file pone.0067551.s001.docx]

Table S1. Development of slow inactivation of DmNa_v_9-1 with or without TipE or TEH1

| Na^+^ channel | τ (msec) | f | n |
| --- | --- | --- | --- |
| DmNa_v_9-1 | 3.58 ± 0.81 | 0.99 ± 0.01 | 10 |
| + TipE | 5.34 ± 0.43 | 0.99 ± 0.01 | 9 |
| + TEH1 | 2.37 ± 0.35 | 0.99 ± 0.01 | 12 |

Development of slow inactivation was fitted by an exponential decay function. Data represents mean ± SEM. τ: time constant, f: fraction of inactivation, n: number of oocytes.
